# Evaluation of Decentralized Solid Waste Management in the Thiruvananthapuram Corporation Area in Kerala, India

**DOI:** 10.7759/cureus.110819

**Published:** 2026-06-14

**Authors:** Deena D S, Nithya G, Himiki S

**Affiliations:** 1 Community Medicine, Sri Venkateswaraa Medical College Hospital and Research Institute, Sri Venkateswaraa University, Chennai, IND; 2 Community Medicine, Kunhitharuvai Memorial Charitable Trust (KMCT) Medical College, Kozhikode, IND; 3 Community Medicine, Sree Gokulam Medical College and Research Foundation, Thiruvananthapuram, IND

**Keywords:** centralized waste management, decentralized, decentralized waste management, solid waste, waste management

## Abstract

Introduction: Solid waste management (SWM) has become a major environmental and public health challenge in urban areas due to rapid urbanization, population growth, and changing consumption patterns. Conventional centralized waste management systems have led to frequent environmental issues. In response to these challenges, many urban local bodies have implemented decentralized SWM that focuses on processing waste closer to the point of generation.

Aim: This study aimed to explore the implementation, stakeholder perceptions, practices, and challenges associated with SWM in the Thiruvananthapuram Corporation area.

Methods: This study was conducted in the Thiruvananthapuram Corporation area of Kerala state among residents and various stakeholders, using in-depth interviews and focus group discussions (FGDs). Thirty in-depth interviews and six FGDs were conducted. The data were transcribed verbatim and free-listed. Domains were identified, coded, summarized, and presented as a summary including quotable quotes.

Results: Disposal of sanitary napkins and diapers was a challenge for most participants, including stakeholders. According to some, a combination of centralized and decentralized waste management systems would be more effective than a single system alone. Integrated waste management technology was being practiced throughout the city. For organic waste, people opted for pipe compost, kitchen bins, and biogas plants, while those who had difficulty managing waste at home opted to hand over their waste to aero bins. The involvement of nongovernmental organizations helped reduce the burden of waste management on local self-governments.

Conclusion: For decentralized systems, public support was lower than for centralized ones. Issues were also due to a lack of operational awareness, poor-quality technology, and inadequate service provision. It has also been found that the agencies failed to supply inoculum to the bins, which can halt the composting process.

## Introduction

With the fastest growing economy and being the most populated country in the world, the annual quantity of waste generated in Indian cities has increased from six million tons in 1947 to 90 million tons in 2009 and is expected to increase to 300 million tons by 2047 [1,2]. The municipal solid waste (MSW) consists of 30%-45% organic matter and 6%-10% recyclables, and the rest is inert matter [3]. According to the 2011 census, of India's 1.21 billion people, 31% live in cities [2]. In India, as per the Central Pollution Control Board, 141,064 tonnes of solid waste were generated per day during 2014-2015. Of the total MSW, approximately 127,531 tonnes per day (TPD) (90%) was collected, but only 34,752 TPD (27%) was processed or treated [4]. Insufficient segregation at the source, collection, and treatment, and unscientific disposal of waste lead to a poor quality of life and environmental degradation [5].

The primary role of a solid waste management (SWM) system is to reduce the adverse effects of solid waste on public health and the environment. The public's demand for sustainable SWM has increased the place on fragile solid waste systems. In an attempt to relieve the increased pressure, several nations opted to shift from centralized to decentralized waste management. In decentralization, responsibilities and authority are transferred toward lower organizational levels. In a centralized system, higher organizational levels, such as the government, hold the decision-making authority [6,7].

Decentralization is the delegation of specific functions by central (i.e., national) government to regional and local (i.e., state/provincial and municipal) governments that are independent of the center within given geographic and functional domains [8].

Rapid population growth, industrialization, and affluent lifestyles have led to a tremendous increase in the quantity of solid waste produced. Improper waste management and disposal are apparent causes of environmental degradation and adverse effects on public health in the developing world [9]. Unscientific incineration, inappropriate waste disposal, etc., have led to greenhouse gas emissions. Anaerobic degradation of waste produces methane gas, which is 21 times more potent than carbon dioxide [10].

Decentralized waste management aims to create a clean, hygienic environment free of garbage, minimize waste disposal, utilize waste at the source to generate energy or useful products, convert organic waste into compost, educate the community and make them aware of their roles and responsibilities, and involve them in SWM. An evaluation of the system can identify any gaps in the implementation, and necessary modifications can be suggested. This will also help in understanding the strengths of the present system that need to be continued, the problem areas that need modification, and some new policies that need to be adopted.

## Materials and methods

This study employed a qualitative research design using thematic analysis to explore stakeholders' perceptions, experiences, and challenges in decentralized SWM in the Thiruvananthapuram Corporation area of Kerala, India. Thiruvananthapuram is the capital city of Kerala state. The municipality came into existence in 1920, and after two decades, it was converted into a municipal corporation on October 30, 1940. The city, which covers 214.86 km^2^, is divided into 100 wards. The population of the city is 957,730 (men: 467,739; women: 489,991), with a population density of 4,457/km² [11]. Until 2011, the city’s waste management system was centralized; currently, it follows a decentralized approach. Ten wards were randomly selected using the lottery method. Participants included key stakeholders relevant to the topic, such as corporation mayors, health officers, health supervisors, health inspectors, managers of various private agencies involved in decentralized waste collection and management, owners of commercial establishments, sanitary workers, members of residents' associations, and community participants residing in the Thiruvananthapuram Corporation area. Participants were selected using purposive sampling to ensure inclusion of individuals with adequate knowledge and experience related to the topic.

Focus group discussions involved 1) health inspectors (one focus group discussion (FGD) with eight participants), 2) community participants (two FGDs with 10 and nine participants each), 3) sanitary workers (two FGDs with eight and nine participants), and 4) residents’ associations (one FGD with 10 participants).

The data were collected using in-depth interviews and FGDs (see the Appendix) (Table 1). To minimize interviewer bias, a semistructured interview guide was used to ensure consistency while allowing participants to freely express their views. Interviewers were trained to use open-ended questions, avoid asking leading questions, and encourage participants to express their views freely. The participants were first contacted and informed about the study. An appointment was then scheduled, the purpose of the study was explained, and they were approached for the interview on a convenient date. Thirty in-depth interviews (corporation mayors, health officers, health supervisors, health inspectors, sanitary workers, managers of private agencies, residents’ association presidents, and community participants) were conducted at locations convenient to the stakeholders and lasted between 45 minutes and one hour, and six FGDs (health inspectors, sanitary workers, flat inmates, residents’ association members, and community participants) were conducted. The number of participants in each FGD ranged from 8 to 10 (for approximately one hour to one hour 30 minutes). All the individuals who consented to participate at the time of approach were included. The interview schedules and FGD guides were developed based on an understanding of the process dynamics of the decentralized SWM system and through informal discussions with administrators from the Swachh Bharat Mission. The schedules were first drafted in English, then translated to the local language and back-translated to ensure accuracy. The interview guide was pilot-tested by performing interviews using a semistructured questionnaire on two health supervisors, one health inspector, and one sanitary worker who were not part of the study.

**Table 1:** Details of stakeholder categories and total IDI IDI: in-depth interview

Stakeholder category	Stakeholder	Number	Total
Policymakers	Corporation mayors	2	2
Program implementers	Health officers	2	2
Field-level functionaries	Health supervisors	2	12
	Health inspectors	4	
	Sanitary workers	6	
Community representatives	Residents of flats/apartments	2	14
	Residents' association	2	
	Community participants	10	
Total	-	-	30

After obtaining consent from the concerned authorities, in-depth interviews were conducted according to the availability of the participants. In-depth interviews with community members were carried out in selected wards. Efforts were made to create a comfortable and nonjudgmental environment during interviews and FGDs to encourage honest responses and reduce social desirability bias. Data collection continued until redundancy was attained. All the interviews and FGDs were audio-recorded. The interview schedule was finalized after consultation with key personnel involved in the system.

Data analysis

Audio recordings from interviews and FGDs were transcribed verbatim. It is then read and re-read thoroughly by the authors to achieve familiarization. Initial impressions were noted down. Coding was performed using a combination of inductive and deductive approaches. Meaningful sentences and phrases were identified, and initial codes were assigned. Similar codes were grouped into categories, which were further organized into broader themes and subthemes. To ensure intercoder reliability and minimize subjective bias, the transcribed data were independently coded by two different researchers. A preliminary codebook was developed based on the interview and FGD guides and on initial readings of the transcripts. Both coders applied the codebook to a subset of transcripts independently. Discrepancies in coding were discussed and resolved through consensus, and the codebook was refined with clearer definitions. The revised codebook was then applied to the remaining data. The final themes and subthemes were clearly defined and presented with supporting verbatim quotes from participants to ensure transparency and credibility.

Data triangulation was achieved by incorporating perspectives from multiple stakeholders across different categories, enabling cross-validation of findings and reducing the risk of single-source bias. Method triangulation, using in-depth interviews (IDIs) and FGDs, provided both individual-level insights and group-level dynamics. The convergence of findings across different data sources and methods enhanced the credibility and validity of results.

## Results

The analysis revealed that the waste management practices among participants were not merely determined by the availability of technologies but were shaped by a complex interaction of household constraints, perceived convenience, and system support mechanisms. In the year 2000, a centralized waste treatment plant was established in the city. However, due to limited capacity and frequent complaints from nearby residents regarding environmental and nuisance issues, the plant was eventually shut down in 2011.

“After the closure of the decentralized plants, waste management became a challenging task for about 2-3 years. During this period, waste was mainly disposed of through landfilling. In some cases, inoculum was added to roadside waste to facilitate composting at the same location." (MY 02-Mayor)

Household-level waste management practices

The participants reported adopting several waste management methods. Common practices included handing over waste to collection units, burying it, or burning it, as well as using decentralized waste management technologies such as pipe composting, biogas plants, kitchen bins, and aerobic bins. The selection of appropriate technology for waste management at each level is influenced by factors such as the availability of space, cost considerations, the quantity of waste generated, and the convenience of use. Households with sufficient space opted for biogas or kitchen bins, whereas pipe composting was preferred by some households with limited space due to its relatively low cost and minimal space requirements. This indicates that structural factors such as space and affordability play a decisive role in technology uptake rather than awareness alone. At the same time, even when technologies were available, continued usage depended heavily on ease of maintenance, suggesting that initial adoption does not guarantee sustained practice (Table 2).

**Table 2:** Themes, subthemes, codes, and representative quotes from interviews and FGDs on decentralized waste management FGDs: focus group discussions

Theme	Subtheme	Codes	Representative quotes and codes
Household waste management practices	Source-level waste management methods	Pipe composting, kitchen bins, aerobins, burning waste, burying waste, handing over to collection units	“Residents select the waste technique based on their specific needs." MY-02 Mayor
	Selection of technology	Availability of space, cost considerations, household waste quantity, convenience	
Benefits of decentralized management	Environmental and economic benefits	Compost production, use of compost in kitchen gardens, fuel generation from biogas	Participants using kitchen bins reported satisfaction as compost was produced within 40-45 days and could be used for gardening
Operational challenges	Technical and maintenance issues	Maggot infestation, foul odor, rodent menace, difficulty in maintenance	Some participants discontinued pipe composting due to foul odor, maggots crawling out through openings, and rodent problems
	Resource and support limitations	High installation cost, lack of repair services, irregular supply of inoculum and coir pith	Participants reported discontinuation of certain technologies due to lack of timely repair and maintenance services
Waste disposal challenges	Plastic waste management	Plastic segregation, monthly collection, burning plastic due to lack of alternatives	Participants reported storing plastic waste until monthly collection, although some burned plastic despite awareness of harmful emissions
	Sanitary waste disposal	Lack of collection for sanitary pads and diapers, burning sanitary waste, alternative disposal methods	Participants reported burning sanitary pads and diapers due to the absence of an organized disposal system
Behavioral and cultural factors	Public attitudes towards waste disposal	Immediate disposal preferences: “not in my backyard" perception	“People often find it difficult to store waste in their homes… many still adhere to the ‘not in my backyard’ concept" - HS01 (Health Supervisor)
	Perception of responsibility	Waste management seen as government responsibility, reluctance to pay service charges	“Many people questioned why they should pay separately for waste management when they are already paying taxes." HO 01 (Health Officer)
Institutional challenges	Governance and monitoring issues	Lack of control over decentralized systems, improper implementation	“A drawback of decentralisation is the lack of adequate control over its implementation." HO 02 (Health Officer)
	Informal waste collection	Unlicensed private waste collectors, illegal dumping	Some private agencies reportedly collected waste from households and dumped it on streets
Suggested improvements	Strengthening waste management system	Increase collection points, weekly plastic collection, systematic waste collection	Participants suggested increasing the number of waste collection points and more frequent plastic waste collection
	Policy and awareness interventions	Surveillance cameras, penalties for littering, public awareness programs	Stakeholders emphasized the need for increased public awareness and stricter enforcement mechanisms

A key stakeholder noted:

“Residents select the waste management technology based on their specific needs and availability of space” (MY02-Mayor)

Perceived benefits of decentralized waste management

Some participants who have been using a biogas plant for waste management reported that sufficient fuel was generated to meet their household needs. Sufficient compost required for the household is generated, thereby reducing external fertilizer purchases and waste reduction at the source. Technologies like kitchen bins were preferred due to their balance between efficiency and usability, as compost formation usually occurs within about 40-45 days. The compost produced can be used in their kitchen gardens, reducing the need to purchase it from external sources. Additionally, kitchen bins are considered esthetically acceptable (Table 3).

**Table 3:** Waste management methods, advantages, disadvantages, and user perceptions reported by participants

Waste management methods	Advantages	Disadvantages	User perceptions
Pipe composting	Requires minimal space; relatively inexpensive; produces compost	Maggots crawling out through openings; foul odor; rodent menace; maintenance difficulties	Initially preferred due to low cost and space requirement, but some users discontinued due to maintenance problems
Kitchen bins	Forms compost within 40-45 days; esthetically acceptable; compost usable in kitchen gardens; reduces need to purchase manure	Presence of maggots and foul odor if waste is not properly managed; liquids must be drained before adding waste	Most users reported satisfaction with the method and continued using for household composting
Biogas plants	Produces fuel for household use; effective treatment of organic waste; environmentally friendly	High installation cost; requires sufficient organic waste; maintenance challenges; limited repair services	Users who generated sufficient waste reported benefits, but others discontinued due to cost and maintenance issues
Aerobins and collection units	Enables collection of both biodegradable and nonbiodegradable waste; useful for households unable to manage waste at home; reduces the burden of household processing; organized disposal	Not available in all wards; residents sometimes travel long distances to dispose waste	Considered convenient where available but accessibility remains a major limitation
Burning waste	Immediate disposal of waste is an easy method when other options are unavailable	Air pollution, harmful emissions particularly when plastic is burned; environmental and health hazards	Some participants resorted to burning plastic or sanitary waste due to lack of alternative disposal options
Burying waste	Simple and low-cost disposal method	Potential contamination; not suitable for all types of waste	Practiced by a few households mainly for organic waste when other methods were not available

Operational challenges in waste management technologies

Despite adopting various technologies, participants reported operational challenges. Users of pipe compost reported problems such as foul odor, the presence of maggots, rodent menace, and difficulty in maintenance. Similarly, kitchen bin users also reported the presence of maggots and foul smell when wet waste, particularly fish waste, was added without proper management. Some participants also mentioned that only properly drained waste could be added to these, which requires additional effort (Table 3).

The biogas plant was perceived as beneficial by some households, as they generated sufficient fuel for domestic use. However, other participants felt that the installation cost was high and maintenance was difficult, particularly when households did not generate sufficient organic waste to sustain the functioning of the plant. Lack of timely repair and maintenance services following installation also contributed to discontinuation of these technologies in some households. The irregular supply of essential materials, such as inoculum and coir pith, negatively affected the proper functioning of waste treatment units (Table 3).

Waste disposal challenges for specific waste

A significant gap was identified in the management of plastic and sanitary waste. Although participants were aware of the harmful effects of burning plastic, many reported doing so because of the lack of reliable, frequent collection systems. Similarly, lack of a formal mechanism for disposal of sanitary waste forced households to adopt unsafe practices. These findings indicate that individual-level behavior is strongly shaped by system-level inadequacies, and environmentally harmful practices may persist even in the presence of awareness.

Plastic and other nonbiodegradable waste was usually stored at home until collection by municipal agencies, which typically occurred once or twice a month. Several participants suggested that plastic waste should be collected weekly to avoid storage difficulties.

Sanitary waste disposal was identified as one of the most significant challenges. The municipal corporation under study did not collect sanitary pads and diapers, which led many participants to burn them. A few participants reported separating cotton from sanitary napkins, flushing them down toilets, and disposing of the remaining material with plastic waste (Table 2).

Behavioral and cultural factors influencing waste management

Cultural attitudes played a substantial role in shaping waste management practices. Many participants expressed discomfort with storing waste at home, reflecting a strong preference for immediate disposal. Additionally, the perception that waste management is primarily the government's responsibility reduced motivation for sustained household-level efforts.

A health supervisor highlighted this cultural preference:

“People often find it difficult to store waste at homes. Culturally, people strongly prefer to dispose of waste immediately after generating it. Many people still adhere to the ‘not in my backyard’ concept.” (HS 01- Health supervisor)

“Many people had earlier questioned why they should pay separately for waste management when they are already paying taxes. They felt that waste disposal is the responsibility of the government." (HO 01-Health Officer)

Institutional- and system-level challenges

Stakeholders reported several institutional challenges affecting decentralized waste management. Limited monitoring and control over waste management practices at the household level were identified as a drawback of decentralized systems.

“A drawback of decentralization is the lack of adequate control over its implementation.” (HO 02 - Health Officer)

Another concern raised was the involvement of unlicensed private agencies that collected waste from households and disposed of it on streets or unauthorized locations. As these agencies provided daily collection services, some residents preferred handing over their waste to them.

Environmental concerns, limited land availability, and public resistance made it difficult to establish new landfill sites (Table 3).

Suggested improvements for waste management

Participants and stakeholders proposed several strategies to improve waste management in the study area. Increasing the number of waste collection points and ensuring regular collection of plastic waste were commonly suggested measures. Many participants recommended weekly collection of plastic instead of monthly collection.

Stakeholders also emphasized the importance of increasing community awareness about available waste management technologies and services. Installation of surveillance cameras in public areas to penalize littering was also suggested as a potential deterrent.

Furthermore, a few key stakeholders recommended that producers should be made responsible for suggesting appropriate disposal methods for products after their use.

“Before introducing any new product into the market, it should be made mandatory for producers to suggest appropriate methods for the disposal of the product after the end of its use.” (HO 02-Health Officer)

Across stakeholders’ groups, there was a consistent perception that neither decentralized nor centralized systems alone were sufficient. Instead, a hybrid model combining both approaches was considered more practical and sustainable (Table 2).

## Discussion

This study provides a comprehensive understanding of waste management practices, challenges, and potential solutions by incorporating perspectives from multiple stakeholder groups, including community members, waste collectors, program supervisors, health officials, and municipal decision-makers. The inclusion of diverse stakeholders enabled triangulation of findings and facilitated a more holistic understanding of the waste management system. The use of both FGDs and IDIs allowed exploration of shared experiences while capturing detailed individual perspectives. The findings provide context-specific evidence that can inform the development of integrated and sustainable waste management strategies in similar urban settings. MSW management is a global problem faced by all developing countries [12].

Despite the benefits, participants reported several operational challenges that limited the sustained use of decentralized technologies. The lack of regular technical support and the irregular supply of essential materials further contributed to the discontinuation of these systems among some users. These findings highlight the importance of continuous technical support and follow-up services to sustain decentralized waste management initiatives. In addition to technologies, the success of decentralized systems depends largely on the interaction among community behavior, institutional support, and system design.

The study also underscores the limitations of focusing exclusively on biodegradable waste management while giving insufficient attention to nonbiodegradable and sanitary waste streams. The persistence of informal disposal practices despite awareness of environmental and health risks suggests that individual behavior is often constrained by the availability of practical disposal alternatives. This highlights the need for policy frameworks that address the entire waste management chain, from segregation and collection to treatment and final disposal.

Another important implication relates to community participation. The findings suggest that public compliance cannot be assumed simply because services or facilities are available. Behavioral norms, perceptions regarding responsibility for waste management, and concerns about storing waste within household premises continue to influence waste management practices. Consequently, awareness campaigns should move beyond information dissemination to foster a sense of shared responsibility and long-term behavioral change.

Stakeholders also pointed out several institutional challenges affecting effective waste management. Limited monitoring and regulation of decentralized systems were considered a drawback, as proper functioning depends on consistent household compliance. The presence of unlicensed private waste collection agencies that improperly dispose of waste was another concern identified by participants. Furthermore, establishing new landfill sites was considered difficult due to land scarcity, environmental concerns, and public opposition. The challenges reported by participants indicate that technological solutions alone are unlikely to achieve sustained behavioral change without adequate technical support, community engagement, and regular monitoring.

Participants and stakeholders suggested several measures to improve management in the study area. These included increasing the number of waste collection points, improving the frequency of plastic waste collection, enhancing public awareness of waste management technologies, and implementing stricter monitoring mechanisms, such as surveillance cameras, to discourage littering. Importantly, most stakeholders emphasized that adopting a hybrid waste management approach combining both centralized and decentralized systems may provide a more practical and sustainable solution for managing urban waste.

In another study, the Delhi Municipal Corporation introduced several Information, Education, and Communication (IEC) materials and carried out campaigns highlighting the benefits of waste segregation and community composting. However, people were found to be unresponsive and unwilling to adopt these practices [13]. It was found that many members of the community were unaware of the various waste management practices and services offered by the government. Even though the authorities claim that sufficient IEC activities were conducted through television, radio, and awareness sessions in residents’ associations, these efforts have failed to reach a large section of the public. This may be due to ineffective campaigns or a lack of reinforcement. Continuous motivation is also necessary to encourage the adoption of such practices [14]. In another study, a pressing need for sustainable approaches to waste management was identified. Although the government has taken several steps in this regard, a more systematic approach is required, along with the use of cost-efficient and cost-effective technologies at various levels wherever possible [15].

India faces a shortage of resources and technological expertise for MSW disposal [16]. In a study on urban waste management in residential areas, the author suggested that legislation and financial powers should be devolved to municipal bodies so they can expand resources and build technical and managerial capacity, thereby minimizing resource wastage [17]. From a policy perspective, the findings support adopting an integrated waste management framework. Most stakeholders favored a hybrid model combining decentralized and centralized approaches. Such a model would allow biodegradable waste to be managed at the source through composting and biogas generation, while ensuring that nonbiodegradable and sanitary wastes are handled through centralized collection, treatment, and disposal systems.

As only a limited number of participants were involved, with limited stakeholder coverage through purposive sampling and a focus restricted to a specific corporation area, the findings may not be generalizable to other municipalities or states. In addition, the responses obtained through interviews and FGDs are subjective, and there is a potential loss of nuance due to translating data from a local language into English. Therefore, a precise quantitative estimate could not be provided; selection and social desirability bias, and lack of objective service indicators like waste volume, collection average, or cost data limited the generalizability of the study.

## Conclusions

The findings indicate that sustainable waste management cannot be achieved through technological interventions alone. Success depends on the alignment of technology, governance, infrastructure, community participation, and broad policy frameworks. Future waste management strategies should, therefore, adopt a systems-based approach that recognizes the interconnected social, behavioral, environmental, and institutional dimensions of waste management.

## Appendices

In-depth interview schedules and FGD guides

In-Depth Interview Schedule-Program Supervisors

1.     Professional background

2.     Roles and responsibilities in waste management

3.     For how long have you been working in this post?

4.     Overview of waste management system

5.     What is the current situation of the waste management of Trivandrum Corporation?

6.     When and how was decentralized waste management introduced?

7.     Pre-implementation

A. Planning

·       How was the planning for the program?

(Who all are involved, what are the things, when, where)

B. Coordination

·       During the implementation, how was the coordination with the

- higher officials

- other officers lower down

- with other sectors

- NGO, voluntary workers, self-help groups

- Facilitators-community leaders, influential people

·       Were the coordination committees formed helpful in the implementation of the program?

·       How did you get communications regarding the system? (Higher officials & lower down)

·       What are the barriers that stand in the way of effective communication regarding the program?

8. Implementation

·       What awareness activities were done during the implementation?

·       Are the funds made available to you enough for the project? What are the sources of funds?

·       Were you able to meet the expectations?

·       What problems have you faced while dealing with the monetary aspects of the program?

·       How can the messages be more effectively communicated to the people?

9. Community participation and behaviour

·       What is your assessment regarding the coverage & conduct of the current system?

10.  General problems & difficulties

·       How do you review the program continuously?

11.  Monitoring and supervision

·       Operational challenges

·       Policy and governance issues

·       What were the general problems and difficulties you faced with the planning and conduct of the program?

12. Management of specific wastes

13. What are your general suggestions for improvement?

14. Any other suggestions/experiences you would like to share/concluding remarks

In-Depth Interview-Haritha Karma Senas/Sanitary Workers

1.     Waste collection practices

2.     Daily work routine and types of waste collected

3.     Awareness of the public regarding decentralized waste management?

4.     From how many houses do you collect waste in a day/week?

5.     What do you do with the collected waste? Do they segregate waste? If not, how do you manage mixed waste?

6.     How is the collected waste transported?

7.     Are there any issues with collection & transportation?

8.     How about the cooperation from the community?

9.      Are you given any remuneration from houses?

10.  Collection from commercial establishments, flats, etc.

11.  What are the shortcomings you see in the program?

12.  Could you please tell some of the problems that you face? Safety and working conditions

13.  Any health problems related to work

14.  What are your suggestions for improving the problem?

In-Depth Interview of Community Participant

1.     Type of household

2.     Awareness regarding decentralized waste management

3.     What types of waste are generated in your house?

4.     Do you segregate your household waste?

5.     If not, how do you deal with household waste?

6.     What method do you follow for each waste type?

7.     Waste collection services

8.     Disposal of specific wastes

9.     What benefits have you experienced from these methods?

10.  What are the problems you face in managing the waste?

11.  Suggestions for improvement

12.  Worries or expectations

FGD Guide-HI/JHI

1.     Reason for introducing decentralised waste management

2.     Awareness

3.     Implementation

4.     Monitoring & supervision

5.     Integration of work

6.     Community effectiveness

7.     Opinion regarding overall success of the programme

8.     General difficulties faced

9.     Suggestions to improve

FGD Community

1.     Community waste practices

2.     Commonly used methods for different types of wastes

3.     Preferred method and why?

4.     Awareness and adoption

5.     Common problems faced in managing waste

6.     Segregation

7.     Satisfied?

8.     Effectiveness of the current waste collection system

9.     General difficulties

10.  Solutions and recommendations
